# Experiences of stigma and HIV care engagement in the context of Treat All in Rwanda: a qualitative study

**DOI:** 10.1186/s12889-023-16752-y

**Published:** 2023-09-19

**Authors:** Charles Ingabire, Dana Watnick, Josephine Gasana, Francine Umwiza, Athanase Munyaneza, Gallican Kubwimana, Gad Murenzi, Kathryn Anastos, Adebola Adedimeji, Jonathan Ross

**Affiliations:** 1 Einstein-Rwanda Research and Capacity Building Program, Rwanda Military Hospital, Kigali, Rwanda; 2 Einstein-Rwanda Research and Capacity Building Program, Research for Development (RD Rwanda), Kigali, Rwanda; 3 Department of Pediatrics, Albert Einstein College of Medicine, Bronx, NY USA; 4 Department of Medicine, Albert Einstein College of Medicine, Bronx, NY USA; 5 Department of Epidemiology and Population Health, Albert Einstein College of Medicine, Bronx, NY USA

**Keywords:** HIV Testing, HIV status visibility, Treat All, HIV stigma, Treatment adherence and compliance, Qualitative Research, Psychosocial Support

## Abstract

**Background:**

‘Treat All’ policies recommending immediate antiretroviral therapy (ART) soon after HIV diagnosis for all people living with HIV (PLHIV) are now ubiquitous in sub-Saharan Africa. While early ART initiation and retention is effective at curtailing disease progression and transmission, evidence suggests that stigma may act as a barrier to engagement in care. This study sought to understand the relationships between HIV stigma and engagement in care for PLHIV in Rwanda in the context of Treat All.

**Methods:**

Between September 2018 and March 2019, we conducted semi-structured, qualitative interviews with adult PLHIV receiving care at two health centers in Kigali, Rwanda. We used a grounded theory approach to data analysis to develop conceptual framework describing how stigma influences HIV care engagement in the context of early Treat All policy implementation in Rwanda.

**Results:**

Among 37 participants, 27 (73%) were women and the median age was 31 years. Participants described how care engagement under Treat All, including taking medications and attending appointments, increased their visibility as PLHIV. This served to normalize HIV and use of ART but also led to high levels of anticipated stigma in the health center and community at early stages of treatment. Enacted stigma from family and community members and resultant internalized stigma acted as additional barriers to care engagement. Nonetheless, participants described how psychosocial support from care providers and family members helped them cope with stigma and promoted continued engagement in care.

**Conclusions:**

Treat All policy in Rwanda has heightened the visibility of HIV at the individual and social levels, which has influenced HIV stigma, normalization, psychosocial support and care engagement in complex ways. Leveraging the individual and community support described by PLHIV to deliver evidence-based, peer or provider-delivered stigma reduction interventions may aid in attaining Treat All goals.

**Supplementary Information:**

The online version contains supplementary material available at 10.1186/s12889-023-16752-y.

## Background

Initiating antiretroviral therapy (ART) soon after HIV diagnosis improves individual clinical outcomes and reduces HIV transmission [1, 2]. Accordingly, in 2015, the World Health Organization recommended ART initiation for all people living with HIV (PLHIV) as soon as possible after diagnosis regardless of immunological or clinical status [3]. This approach, known as Treat All, has been adopted globally and in Sub-Saharan Africa (SSA) most PLHIV are now initiating ART soon after diagnosis [4]. Despite the emphasis on early ART initiation, an emerging literature has described challenges in early engagement in HIV care under Treat All [5, 6] and questions remain about how to optimally implement this program.

In other contexts, HIV-related stigma has been identified as a major barrier to engagement in HIV care, acting through multiple mechanisms including *experienced stigma* (actual experiences of discrimination directed towards PLHIV), *anticipated stigma* (the degree to which PLHIV expect they will experience discrimination from others), and *internalized stigma* (the degree to which PLHIV endorse negative beliefs and feelings related to HIV) [7]. There is also an extensive literature describing the negative impact of HIV-related stigma on HIV testing [8], care initiation [9], attendance at appointments [10] and adherence to ART [11, 12]. Nonetheless, few studies have examined experiences of HIV-related stigma in the Treat All era [12–14].

Health care delivery changes under Treat All—including rapid ART initiation, larger numbers of PLHIV on treatment and streamlined care may directly or indirectly affect HIV-related stigma in both positive and negative ways. While Treat All implementation may reduce stigma through normalization of HIV testing, increasing solidarity among PLHIV, and maintenance of good health associated with ART [15], increasing engagement in care and use of ART has also been found to result in harmful effects of status disclosure including subsequent discrimination [16]. Initial studies examining the impact of treatment expansion on stigma have reported mixed results. For instance, a longitudinal, population-level analysis of data from 18 African countries demonstrated that increases in ART coverage were associated with decreasing percentages of people reporting HIV related stigma [17]. However, data from the large HPTN 071 PopART study in South Africa and Zambia found that widespread HIV testing under Treat All did not reduce participants’ anticipated stigma for testers and fellow community members in high-prevalence communities [13] and had an overall limited impact on HIV-related stigma among PLHIV, people who are HIV-negative, and health workers [18]. Two other qualitative studies described stigma as a barrier to uptake of and engagement in HIV care among PLHIV initiating ART under Treat All [5, 6], yet PLHIV participating in a Treat All pilot program in eSwatini endorsed the potential of widespread, early ART to reduce stigma by avoiding manifesting HIV-related symptoms [14].

Few studies have examined experiences of HIV-related stigma and its impact on engagement in care in the context of Treat. To better understand these relationships, we conducted a qualitative study of PLHIV in Rwanda, one of the first countries in SSA to implement Treat All.

## Methods

### Study design and setting

This study reports on qualitative data originally collected to understand barriers and facilitators to uptake of ART in the context of national implementation of Treat All in Rwanda [19]. The relevance of stigma to ART uptake inductively emerged from early analyses of those data, therefore deserving further exploration, as presented in this manuscript. Semi-structured in-depth qualitative interviews lasting 60–90 min were conducted at two health centers (HCs) providing HIV care in Kigali, Rwanda from September 2018 to March 2019. Both HCs are located in an urban area where HIV prevalence is higher (4.3%) than the national prevalence (3%) [20]. Both HCs started providing Treat All services from July 2016 when the Government of Rwanda launched the countrywide program to provide ART to every person diagnosed with HIV as soon as possible, preferably within one week of diagnosis [21].

### Participant recruitment

We recruited a sample of PLHIV via health care providers at each HC who identified potential research participants and linked them to study staff for eligibility screening. Inclusion criteria were (1) being ≥ 18 years old; (2) living with HIV; and (3) receiving/had received care from study HCs. We purposefully recruited younger participants (ages 18–24), whose adherence to ART is frequently poorer than younger or older PLHIV [22, 23]. We also preferentially recruited participants who had missed at least one scheduled appointment over the year prior to study enrollment. We aimed to include at least one third of younger participants and one third of participants with poor engagement in care. Participants were compensated for transport and their time. Participants were recruited until thematic saturation occurred, generating no new findings.

### Data collection and quality

We developed a semi-structured interview guide informed by the socioecological model [24] and piloted it with research staff not involved in the current study to ensure appropriateness and length [19]. The interview guide explored individual barriers and facilitators, at family or community level, health center level, and policy making level. Interviews were conducted in Kinyarwanda language by two Rwandan female research staff (FU, JG) with specialized training in qualitative data collection and analysis. Interviewers had no prior relationship with participants. All interviews were conducted in a private room, with the presence of study staff only, to ensure recording quality and participant privacy, audio-recorded, and later transcribed and translated into English. During interview administration, FU and JG were both present: one conducted the interview and the other took notes. Interview quality was monitored by CI, observing early interviews and providing feedback with the data collection team and the Principal Investigator through weekly conference calls. Informed consents were kept in a locked cabinet, transcripts were de-identified, and audio-recordings were destroyed after transcription.

### Data analysis

We used a grounded theory approach to data analysis, using the constant comparative method, comparing data episodes, codes, themes and concepts. We chose grounded theory because it is an inductive method that is appropriate when little is understood about a phenomenon (i.e., manifestations of stigma in the context of Treat All policy), and when new theory is desired to help explain processes and mechanisms of behavior [19, 25, 26]. In the first analytic step, three members of the analysis team (CI, FU, JG) created a case-based memo for each interview. Analysts then identified all relevant excerpts related to stigma for data reduction. Analytic memos were developed to track theme and pattern development and to assemble higher-order concepts before, during and after weekly analysis team discussions. Simultaneous to case-based and analytic memo construction, the team began an iterative codebook development process, which involved identification of early ‘repeating ideas,’ using constant comparisons of findings across transcripts to help refine and define codes [27]. This exercise resulted in an initial codebook that was entered into Dedoose, an online tool used to help manage and analyze qualitative data [28]. Each of the first 6 transcripts was initially coded by 2 analysts (CI, FU, or JG) using the preliminary codebook, discussing agreements and discrepancies. A senior investigator (DW) reviewed coded transcripts and facilitated code refinement. The analytic team then applied the final codebook to all transcripts with each transcript coded by at least 2 analysts. Grouped excerpts were further examined within each code and sub-code to develop themes and concepts and relationship among them. Through iterative group discussion, which included returning to the data to examine negative cases, we developed a preliminary conceptual framework. Finally, in late phase analysis, we also compared our emergent conceptual framework with Goffman’s existing stigma theory to identify similarities and differences [29–31]

### Ethical considerations

The Rwanda National Ethics Committee (RNEC approval number 254/RNEC/2018) and the Institutional Review Board of the Albert Einstein College of Medicine (Einstein IRB approval number 2017–8234) approved the study, which was conducted according to the principles expressed in the Declaration of Helsinki and is reported in accordance with Consolidated Criteria for Reporting Qualitative Research (COREQ) guidelines [32]. An additional file shows more details (see Additional file 1). Written informed consent was also obtained from all participants prior to study enrollment.

## Results

**Fig. 1:**
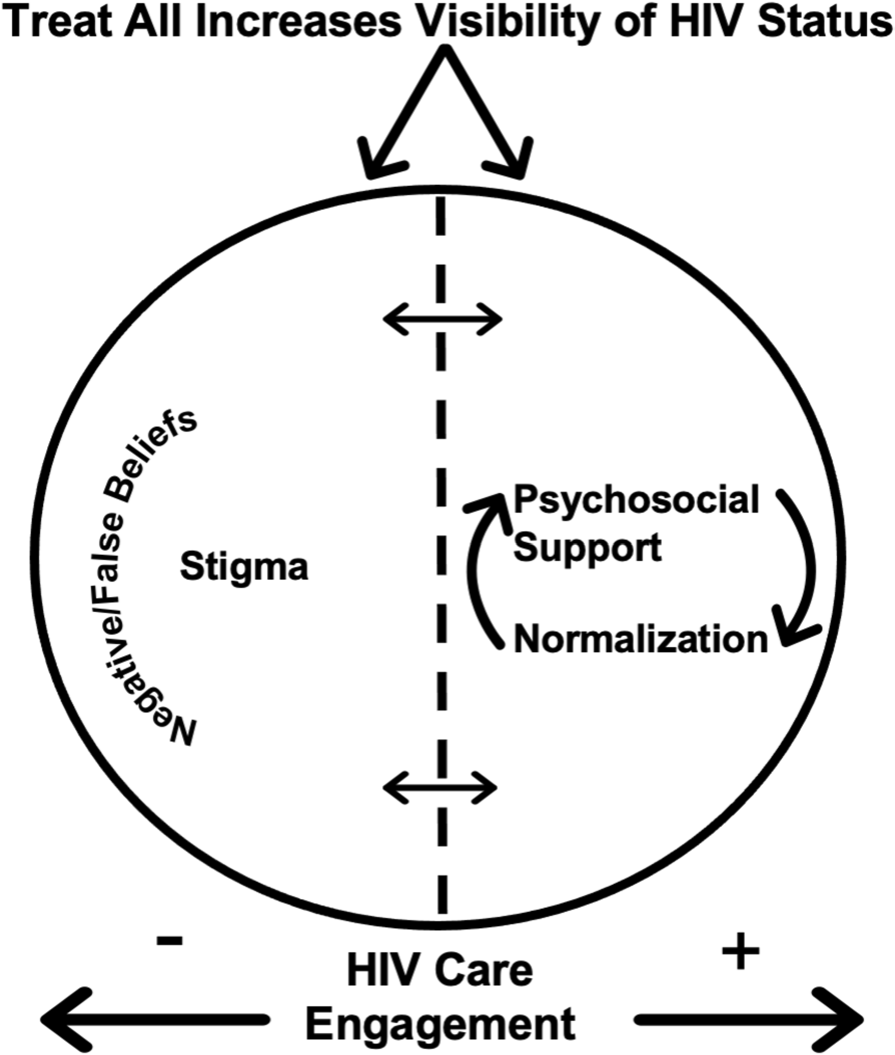
Conceptual framework of stigma and HIV care engagement in the era of Treat All

We interviewed 37 participants, of whom 27 (73%) were women and 15 (40%) were aged 18 to 24 years (Table 1). Four major themes related to the impact of Treat All implementation emerged: (1) Treat All normalizes HIV and ART; (2) visibility of HIV status feeds stigma; (3) enacted and internalized stigmas are barriers to care engagement and (4) psychosocial support remediates stigma-related barriers to care. Figure 1 delineates a summary of the findings, which are elaborated in the sections below.

**Table 1:** Demographic characteristics of study participants (*N* = 37)

Characteristics	n	Percentage
Gender		
Female	27	73%
Male	10	27%
Age		
18–24	15	40%
≥ 25	22	60%
Time of diagnosis		
Prior to Treat All	7	19%
After Treat All	30	81%
Median time from ART eligibility to initiation	2 months	-
Median time from ART initiation to interview	18 months	-
Missed ≥ 1 appointment in the last 12 months	15	40%
18–24	4	27%

### Treat all normalizes living with HIV and initiating ART

Participants described how engaging in early HIV care offered a pathway to a healthy future. Some described their initial skepticism of this possibility, grounded in deeply-held beliefs that an HIV diagnosis implied certain death:

*“When they give you a result that you are HIV positive you feel like you are dying. When the healthcare providers are providing you with counseling that you will live longer, you don’t even understand that. You start to count years and think that you are dying in 2 years or in 5 months. But when you start medication, you don’t feel weakened, you don’t stop working; that is when you start to accept that life continues as normal.”* Participant #21 (33-year old male).

Yet through care experiences and messaging from health care providers, participants began to understand the physical and mental health benefits of treatment. Participants shared how early ART initiation allowed them to live normally in different arenas of life including delivering healthy babies, completing school, having a good job, and providing for their families. Many times, seeing other healthy PLHIV at the HC allowed participants to reflect on the possibility that they too could achieve or maintain good health, and helped them normalize life with a chronic disease:

*“Because of talking with people who told me that they had been living with HIV for 15 or 20 years and meeting with people at the health center, you realize that that person is healthy and you also think that you will be healthy as well.”* Participant #21 (33-year old male).

Participants also appreciated that initiating ART earlier in the disease process could interrupt or prevent physical manifestations of advanced HIV. Some described how they were more likely to take medication in order to prevent the physical signs of HIV, like this participant who said:

*“The medications prevent me from developing opportunistic infections, I physically look good without any problem because if you don’t take medication, as it has to be taken, you can sometimes develop opportunistic infections where people can see and guess what you suffer from.”* Participant #26 (23-year old male).

### Increased visibility feeds anticipated stigma

While some heralded the benefits that came with the normalization of living with HIV and the ability to look healthy, there remained pressing concerns that the logistics of care engagement could increase the visibility of their status. Participants observed that the number of people collecting antiretroviral drugs at the health center increased with Treat All implementation, leading to longer waiting times and likelihood of being seen by someone they knew. One said:

*“They [neighbors] saw me at the HIV service at the health center and they told neighbors that I am also infected with HIV. That is the challenge that shocked my heart.”* Participant #34 (34-year old male)**.**

This increase in visibility catalyzed an increase in anticipated stigma, such that participants increasingly feared what might happen if their status became more obvious. Participants specifically identified the stigma associated with waiting in a designated HIV section of the health center, where their HIV status was obvious to all. One said:

*“People who come seeking general health services can see you entering the HIV program room and they ask information about the services provided in those rooms, then they get information that those are the rooms for the HIV program. That is how they guess that a person has HIV, and go out to spread it [this information], even far away.”* Participant #2 (27-year old female).

The increased risk of inadvertent status disclosure was especially difficult for newly diagnosed patients, who were required to come to the health center for frequent appointments while simultaneously coping with the realities of a new HIV diagnosis.

In response to this increased visibility, participants described various ways they attempted to minimize status disclosure, including using alternative routes to reach the health center; arriving at less crowded hours; hiding when encountering people they knew; or even seeking care at health centers far from their homes. One participant stated:

*“They asked me if I could get medication from that health center … I told them that I wanted to go to [a distant] health center. I trust this health center because there are no people who knew me before I was diagnosed with HIV.”* Participant #29 (35-year old female).

Some went further to avoid disclosure, skipping appointments, avoiding visits from health care workers, neighbors, and friends, or discontinuing ART altogether:

*“There are times I missed my appointment to pick up medication because I saw a person at the health center who knew me while that person does not know that I live with HIV. I missed the appointment and came back the next day.”* Participant #18 (24-year old male).

*“When I started medication, I tried to avoid people …Sometimes your friend could come to the house, get into the house by surprise and could see your medication. I started to distance myself from people from the time I started medication. I started to live a lonely life and when I left the house, I padlocked the house. I started to be choosy about guests to avoid people knowing that I was infected. During the prior 4 months I told you, I had them [the pills] but did not take them because I didn't want people in my neighborhood to learn that I was infected with HIV. Even when I had a guest, I didn’t take medication.”* Participant #17 (35-year old female).

Participants additionally anticipated stigmatizing reactions from intimate partners, friends, or close relatives if they actively disclosed their status. This anticipation deepened internal struggles regarding when, how, and with whom to confide in:

*“The husband continued to wait expecting me to tell him something but I didn’t tell him my status. I was also afraid to disclose it to my child. I kept hiding my medications, till now; I have never disclosed my status to any person except one woman we lived together that was also infected with HIV. That is the only one I disclosed to.”* Participant #35 (30-year old female).

In its most extreme form, the fear of disclosure and anticipated stigma led to suicidal ideation:

*“I was wondering what would happen if my mother and my other brothers learned that I was infected with HIV at my age. I felt dishonored and thought that instead of people saying that I died of HIV, I was going to kill myself.”* Participant #20 (24-year old female).

### Enacted and internalized stigmas are barriers to early engagement in care

Participants described experiences of discrimination by health care providers, family and community members that made it challenging to engage in HIV care. These stigmatizing experiences left them feeling ashamed, harassed at their places of work, and rejected by family members. In some circumstances, their HIV status was disclosed to employers or family members without their consent. One woman took a job as a nanny but experienced stigma from her health care providers:

*“They [healthcare providers] kept telling my employer that I might hurt the baby with an infected sharp object. They kept telling my employer to be aware that I was infected with HIV…. I was sad too. I decided to never trust any person.”* Participant #29 (35-year old female).

In some cases, participants reported rejection from close family members and romantic partners. In addition to the emotional strain this caused participants, it also created anxiety about being cast out from the family home and experiencing financial strain on top of homelessness. One participant experienced marital conflict and being treated poorly by her husband:

*“I was in trouble with my spouse who was asking me where I got HIV. Sometimes my spouse refused me to take medication.”* Participant #16 (33-year old female).

Discrimination in the community and even at the health center contributed to high levels of internalized stigma and low self-worth. One participant described how:

*“In a month, I had hated myself, I was thinking about it. I hated myself feeling that I was not a human being. I felt I was nothing. When I was walking with someone, I felt like I was walking alone.” *Participant #33 (23-year old female).

Frequently, this internalized stigma was driven by negative perceptions, false beliefs and outdated information about HIV that persisted in society. Some of these beliefs included: those infected with HIV experience an ugly or gruesome death; once diagnosed you die immediately; the inevitability of giving birth to an infected child, among others. These false beliefs, often internalized prior to HIV diagnosis, led to attitudes that could negatively impact engagement in care such as feelings of despondency, and even suicidality. One participant stated:

*“In the family they sometimes talk about HIV negatively and the people living with HIV become afraid to disclose their status to family members because they know how they may react. At home they used to say that it is a sacrilege to be infected with HIV. I remember that once I went to visit other children and when I arrived home my uncle beat me because I went to a home where PLHIV lived…I thought to commit suicide when I arrived at home.”* Participant #17 (35-year old female).

*“When the healthcare provider told me the result, I felt that I was going to die even before I reach the tarmac road to take a bus……I decided to kill myself because I thought also I would die losing my skin and having larvae out of my body. I thought to kill myself so that I could die without anyone knowing that I was infected with HIV.”* Participant #20 (24-year old female).

### The role of psychosocial support in counteracting stigma-related barriers to care

Despite the challenges related to increased potential for unwanted status disclosure, the increased visibility offered additional opportunities for participants such as engaging in counseling from healthcare providers, sharing experiences with community members, and receiving emotional and logistical support from friends and relatives that allowed them to counteract stigma-related barriers to care. This psychosocial support was particularly salient early in the treatment process, when participants experienced high levels of anticipated stigma. They expressed that these unexpected and often surprising expressions of support from their social and healthcare networks was one of the main benefits of initiating care early. Participants said that early counseling and emotional support helped them. For example:

*“When I went to the health center and was diagnosed with HIV, I thought that if someone is infected with HIV, they die immediately. But they explained to me a person infected with HIV does not die immediately; instead, life continues. That continued to give me hope that if I take medication, life will continue.”* Participant #23 (23-year old female).

Specifically, support from health care providers was instrumental throughout critical steps in the HIV care continuum, both overcoming the initial shock of an HIV diagnosis as well as later engagement in care. As one participant noted:

*“If I had not had a healthcare provider to provide me with counseling, I would not have been able to accept my status. I told you before that I felt worthless; access to healthcare providers made me regain morale.”* Participant #14 (23-year old female).

Participants expressed many benefits of early emotional and logistical support from friends and relatives, including concrete actions such as accompaniment to the health center and picking up medication on their behalf, reviving hope for the future, adherence support, and moral support. This participant disclosed to a relative immediately after diagnosis:

*“I immediately called my sister, she helped me to continue to be patient. When she arrived, she told me that there was no other choice except to be patient. My sister assured them that there would be no reason that she could not stay close to me and added that she would be supportive and she would come to pick my medications if I didn't have time. She helped me a lot, she bought fruits for me, made juice for me; and progressively became familiar with medications to the extent I felt I could swallow them with water. I wondered if my child would be HIV negative because I didn’t understand how I could give birth to an HIV negative child while I was infected with HIV. My sister assured me that it could be possible because there are many people who are HIV positive but deliver HIV negative children. She continued to stay close to me, I took medications properly on time, and those thoughts ended.”* Participant #31 (23-year old female).

The organization of care under Treat All also allowed for support from others living with HIV. In a setting with additional patients accessing the health center, less time with clinicians, and reluctance to disclose one’s HIV status to family or friends, peer support could be of particular importance. One participant described how:

*“[The] Treat All program increases our morale because when you are together with others you don’t feel lonely. You meet together and feel happy. You share advice; there are many [pieces of] advice when you meet. It helps a lot and makes us feel very committed to take medications. If you are alone, you can feel ashamed to go there alone. But when you meet there as a big group, you talk and share advice.”* Participant #29 (35-year old female).

In contrast to participants who experienced rejection and discrimination from family and community members, those who received support from members of their social networks noted their critical role in helping to overcome internalized stigma and remain engaged in care. One stated:

*“They found that I was infected with HIV. I went out of the room feeling ashamed and thought about jumping off of the building because it was a tall building. I thought it was the end of my life. My older brother told me to be patient, it was not the end of my life and promised me support from the family for any need I would have.”* Participant #18 (24-year old male).

## Discussion

This is one of the first studies that examined how HIV-related stigma can impact HIV care engagement under Treat All policies. A defining feature of Treat All is its temporal focus on initiating HIV care earlier to prevent disease progression and transmission of HIV to others. In the Treat All era, instead of HIV-related illnesses or AIDS marking a person’s entry into care, most are now asymptomatic at HIV diagnosis, offering opportunities for life-saving care and good health. Nevertheless, far-reaching policies like Treat All can offer both massive improvements to individual and public health, but may create inadvertent secondary effects. One of the ways that Treat All aims to achieve its goals is through reframing HIV as a treatable chronic illness rather than the death sentence it once was prior to the advent of ART [33], and to raise consciousness and decrease marginalization of PLHIV.

In our study, participants described the ways in which their individual HIV status was visible from the early days of their diagnosis, which had both positive and negative consequences vis à vis stigma and their desire or ability to engage in care. This increased visibility manifested two counteracting pathways to HIV care engagement versus non-engagement. On one side, the visibility offered the opportunity to normalize a future and a healthy life by engaging in HIV care. In this pathway Treat All demonstrated the potential to counteract stigma through normalizing a life with HIV and engagement in HIV services. For example, starting HIV medication early in the disease process allowed PLHIV to remain healthy and led to interactions with other PLHIV, including peer support groups, that made them appreciate how living with HIV can be normal, in turn counteracting internalized and anticipated stigma and making it easier to engage in care. Similar findings were observed by Horter, et al., in a study of PLHIV engaging with Treat All care in eSwatini [14]. Several mechanisms may underlie this observed normalization: the role of ART as a mechanism for maintaining health, thus living normal and productive lives and avoiding status disclosure through ill health, has been previously described [33, 34]. Additionally, Camlin et al., suggested that increased visibility at clinics as more PLHIV initiate care under Treat All could lead to positive experiences with status disclosure and solidarity with other PLHIV, in turn decreasing anticipated stigma [15].

In a second pathway, however, participants described related fears and concerns about having their HIV status exposed and our results suggest that the prospect of status disclosure as a result of engaging in HIV care under Treat All can worsen anticipated and internalized stigma. This anticipated stigma was not unfounded, as participants sometimes experienced discrimination in care facilities and communities, in turn driving internalized stigma and sometimes leading to early disengagement from care. Participants described going to great lengths to avoid exposure through taking medication or being seen at the health center. Importantly, although few participants reported enacted stigma, those who did often had their first stigma experience at the health center, which can be particularly detrimental to ongoing engagement in HIV care as has been shown in different studies [5, 12]. Consequently, some participants disengaged from some aspects of care, such as not taking ART and skipping appointments, behavior observed in other studies examining engagement in HIV care in the Treat All era [34, 35]. Indeed, both qualitative and mechanistic studies under Treat All have reported direct and independent relationships between anticipated stigma or stigma avoidance and ART non-initiation or discontinuation [12, 36, 37]. While one of the stated goals of early ART for all PLHIV under Treat All is to minimize pre-ART loss from care [3], our results suggest that pressure to engage in care soon after diagnosis can have the unintended consequence of worsening stigma and negatively impacting care engagement. To maximize the benefit of Treat All, additional stigma reduction efforts within healthcare setting specifically should be studied as they may decrease disengagement in care.

The process of moving from stigmatized to normalized is not easy under Treat All, as PLHIV are asked to start ART immediately, often while still coping with their diagnosis. The perceived cost of disclosure may outweigh the benefits of ART, compared to earlier paradigms where advanced disease was a criterion to start HIV medication [36]. However, participants who accepted the exposure associated with attending health center appointments and taking medications were able to access psychosocial support from healthcare providers, friends, and relatives which was beneficial in counteracting stigma and improving engagement in care overall. Previous studies have shown that lack of psychosocial support increases stigma and disengagement in care while the availability of psychosocial support by health care providers, family, and friends reduces stigma and improves engagement in care [34, 38–41]. For maximizing effectiveness of Treat All policies, HIV care programs will need to devote resources towards broad stigma reduction efforts, particularly early in the course of treatment, that also enhance supportive psychosocial networks.

Our findings show that individual counseling and participation in support groups can positively impact PLHIV through supporting and normalizing a healthy life with an HIV diagnosis. This normalization of HIV through social interactions together with the routinization of HIV care, can contribute greatly to the process of HIV normalization and thus decrease the negative effects of an HIV diagnosis on different functioning areas of life such as education, employment, or having children or a family. Health care providers and families, through the diverse support they extend to newly diagnosed PLHIV, are key to normalizing experiences of living with HIV. Psychosocial support plays an important role under Treat All as, in addition to emotional burden caused by the infection, PLHIV are requested to engage in care which appears to be demanding given the emotional status they are in. Prior studies have shown that psychosocial support helps people infected with HIV to cope with its different consequences in different areas of life [13, 42, 43]. However, the fear of disclosure can limit access to psychosocial support which can result in poor engagement in care, suicidal ideation, poor treatment outcomes further hindering normalization of HIV infection, treatment, and ultimately, well-being of those living with HIV [34, 35, 44, 45].

We observed high levels of anticipated and internalized stigma among participants in this study, driven by fear of inadvertent status disclosure and fueled by negative perceptions and false beliefs about HIV that remain prevalent in Rwandan society. Our results suggest that fully realizing the potential of Treat All will require interventions that reduce stigma and facilitate normalization, particularly in the early period after HIV diagnosis. Psychosocial support as a major facilitator of the process of moving from stigmatized to normalized should be given particular attention and importance given the short time-frame to start medication after diagnosis and relative health of PLHIV. The positive role of psychosocial support for PLHIV from multiple levels of society (from family members, healthcare workers, and other PLHIV) in counteracting people’s HIV stigma experiences suggests that this may be a promising area for intervention [42, 43]. Additionally, structural interventions that reduce visibility for people living with HIV, such as fewer appointments under differentiated service delivery models, should be explored as potential avenues for stigma reduction [46]. Moreover, stigma has different dimensions which is why multiple strategies are necessary, for example training providers on cognitive behavioral interventions and stress-reduction approaches, implementing problem-solving therapy, peer-led skills building activities, and utilizing widespread stigma-reduction campaigns such as U = U or promoting HIV status disclosure [47–49].

Several limitations of this study are worth noting. Participants had engaged in some level of care, so they may have engendered more positive perspectives on care engagement than those who were not in care. Moreover, willingness to participate in research may reflect an overall lower degree of stigma. We therefore may not have captured the perspectives of PLHIV who are too stigmatized to even come to the health center. This study was conducted in two urban health centers in a country with a highly successful HIV program [20]. Consequently, these findings must be interpreted with this demographic and treatment context in mind, therefore our proposed model can provide a useful lens to examine how varying forms of stigma may be experienced and subsequently impact care engagement differently. Stigma-reduction efforts in the community, including media campaigns promoting U = U, peer support groups, and others may also help reduce persistent misperceptions, societal stigma, and enacted stigma towards PLHIV.

## Conclusion

We found that Treat All can have a normalizing effect on living with HIV and improve engagement in care, but the increased HIV status visibility that results from HC interactions and taking ART can also reinforce stigma, making the process of normalization challenging. Participants’ anticipated stigma about and actual experiences of HIV status exposure made them reluctant to engage in care, but also provided opportunities to receive psychosocial and emotional support from families, communities, and health care workers. Our findings support implementation of interventions that reduce multiple types of stigma as well as those that bolster psychosocial support at the individual, family, community, and HC levels may help to achieve the promise of Treat All.

### Supplementary Information

**Additional file 1.** Consolidated criteria for reporting qualitative studies (COREQ) 32-item checklist.

**Additional file 2.** Example questions from interview guide and socioecological levels.

## Data Availability

Relevant excerpts are included in the body of the manuscript. Full transcripts are not publicly available as they contain sensitive information, which, though de-identified include personal narratives that could result in identification of individuals, and because permission for public dissemination of raw data was not solicited from the Rwanda National Ethics Committee. De-identified data that support the findings of this study may be made available for researchers who meet the criteria for access to confidential data upon reasonable request. For data inquiries, please contact the Administrator of the Rwanda National Ethics Committee, at info@rnecrwanda.org.
