# Supplementary material for: Experiences of stigma and HIV care engagement in the context of Treat All in Rwanda: a qualitative study
Source: BMC Public Health. 2023 Sep 19;23:1817. doi: 10.1186/s12889-023-16752-y (PMC10507909; doi:10.1186/s12889-023-16752-y)
Supplement: Supplementary file 1 — Additional file 1. Consolidated criteria for reporting qualitative studies (COREQ) 32-item checklist. [file 12889_2023_16752_MOESM1_ESM.docx]

**Consolidated criteria for reporting qualitative studies (COREQ) 32-item checklist**

| Checklist item | Description | Reporting location |
| --- | --- | --- |
| Domain 1: Research team and reflexivity | | |
| 1. Interviewer | Which authors conducted the interview? | Methods, Parag. 3 |
| 2. Credentials | What were the interviewer’s credentials? |  |
| 3. Occupation | What was their occupation at the time of the study? |  |
| 4. Gender | Was the interviewer male or female? |  |
| 5. Experience and training | What experience or training did the interviewer have? |  |
| Relationship with participants | | |
| 6. Relationship established | Was a relationship established prior to study commencement? | Methods, Parag. 3 |
| 7. Participant knowledge of interviewer | What did participants know about the researcher? |  |
| 8. Interviewer characteristics | What characteristics were reported about the interviewer? |  |
| Domain 2: Study design | | |
| Theoretical framework | | |
| 9. Methodological orientation and Theory | What methodological orientation was stated to underpin the theory? | Methods, Parag. 4 |
| Participant selection | | |
| 10. Sampling | How were participants selected? | Methods, Parag. 2 |
| 11. Method of approach | How were participants approached? |  |
| 12. Sample size | How many participants were in the study? | Methods, Parag. 2 |
| 13. Non-participation | How many people refused to participate or dropped out? | Result, Parag. 1 |
| Setting | | |
| 14. Setting of data collection | Where was the data collected? | Methods, Parag. 1 |
| 15. Presence of non-participants | Was anyone else present besides the participants and researchers? | Methods, Parag. 3 |
| 16. Description of sample | What are important characteristics of the sample? | Results, Parag. 1 |
| Data collection | | |
| 17. Interview guide | Were questions, prompts, guides provided by the authors? Was it pilot tested? | Methods, Parag. 3 |
| 18. Repeat interviews | Were repeat interviews carried out? If yes, how many? | Methods, Parag. 3 |
| 19. Audio/visual recording | Did the research use audio or visual recording to collect the data? |  |
| 20. Field notes | Were field notes made during and/or after the interview or focus group? |  |
| 21. Duration | What was the duration of the interviews or focus group? | Methods, Parag. 1 |
| 22. Data saturation | Was data saturation discussed? | Methods, Parag. 2 |
| 23. Transcripts returned | Were transcripts returned to participants for comment and/or correction? | No |
| Domain 3: analysis and findings | | |
| Data analysis | | |
| 24. Number of data coders | How many data coders coded the data? | Methods, Parag. 4 |
| 25. Description of the coding tree | Did the authors provide a description of the coding tree? |  |
| 26. Derivation of themes | Were themes identified in advance or derived from the data? |  |
| 27. Software | What software, if applicable, was used to manage the data? |  |
| 28. Participant checking | Did participants provide feedback on the findings? | No |
| Reporting | | |
| 29. Quotations presented | Were participant quotations presented to illustrate the themes/findings? Was each quotation identified? | Results, all paragraphs |
| 30. Data and findings consistent | Was there consistency between the data presented and the findings? |  |
| 31. Clarity of major themes | Were major themes clearly presented in the findings? |  |
| 32. Clarity of minor themes | Is there a description of diverse cases or discussion of minor themes? |  |
