# Supplementary material for: Experiences of stigma and HIV care engagement in the context of Treat All in Rwanda: a qualitative study
Source: BMC Public Health. 2023 Sep 19;23:1817. doi: 10.1186/s12889-023-16752-y (PMC10507909; doi:10.1186/s12889-023-16752-y)
Supplement: Supplementary file 2 — Additional file 2. Example questions from interview guide and socioecological levels. [file 12889_2023_16752_MOESM2_ESM.docx]

| **DOMAINS OF CARE** | **QUESTION** | **LEVEL OF SOCIOECOLOGIC MODEL** |
| --- | --- | --- |
| **Experiences of HIV diagnosis** | 1. Tell me about when you were first diagnosed with HIV | Individual |
| **Experiences initiating ART** | 1. Tell me about your experience first starting medications? How did you feel physically and emotionally after starting medications? | Individual |
|  | 1. Were there certain problems or challenges you had that made it difficult to start medication? What were they? | Individual, social network, institutional |
|  | 1. Were there certain things that made it easier to start medication? What were they? | Individual, social network, institutional |
|  | 1. How did the environment at the clinic/health center affect the process of starting medications? | Institutional |
|  | 1. Are there other things that the health center or government could do to make it easier to start taking medication? What are they? | Institutional, societal |
| **Experiences adhering**  **to ART** | 1. Tell me about any difficulties you have experienced in continuing to regularly take medication for HIV | Individual, social network, institutional |
|  | 1. Are there certain things that make it easier to stay on medications? What are they? | Individual, social network, institutional |
|  | 1. Are there other things you think the health center or government could do to make it easier to stay on medications? What are they? | Institutional, societal |
| **Experiences adhering**  **to appointments** | 1. Tell me about any difficulties you have had in continuing to regularly come to appointments at the health center for your HIV care | Individual, social network, institutional |
|  | 1. Are there certain things that make it easier to stay in care at the health center? What are they? | Individual, social network, institutional |
|  | 1. Are there other things you think the health center or government could do to make it easier to attend appointments? What are they? | Institutional, societal |
| **Perceptions of Treat All and of health care delivery** | 1. The government of Rwanda has decided that every person diagnosed with HIV should receive ART, and that they should start medications as quickly as possible after diagnosis. How do you feel about this? | Societal |
|  | 1. Are there parts of your HIV care that you think are missing or insufficient? What are they? | Institutional |
|  | 1. Are there parts of your HIV care that you think are unnecessary? What are they? | Institutional |
